# The circulating exosomal microRNAs related to albuminuria in patients with diabetic nephropathy

**DOI:** 10.1186/s12967-019-1983-3

**Published:** 2019-07-22

**Authors:** Hyoungnae Kim, Yun-Ui Bae, Jin Seok Jeon, Hyunjin Noh, Hyeong Kyu Park, Dong Won Byun, Dong Cheol Han, Seongho Ryu, Soon Hyo Kwon

**Affiliations:** 1 Department of Internal Medicine, Soonchunhyang University Seoul Hospital, Seoul, South Korea; 2 Hyonam Kidney Laboratory, Soonchunhyang University Seoul Hospital, 59 Daesagwan-ro, Youngsan-gu, Seoul, South Korea; 3 Soonchunhyang Institute of Med-bio Science (SIMS), Soonchunhyang University, Chonan, South Korea; 4 Soonchunhyang Institute of Med-bio Sciences (SIMS) and Laboratory of Pathology, Department of Medicine, Soonchunhyang University, Chonan, 336-745 South Korea

**Keywords:** Albuminuria, Diabetic nephropathy, Exosome, MicroRNA

## Abstract

**Background:**

Diabetic nephropathy (DN) is associated with high risk of cardiovascular disease and mortality. Exosomal microRNAs (miRNAs) regulate gene expression in a variety of tissues and play important roles in the pathology of various diseases. We hypothesized that the exosomal miRNA profile would differ between DN patients and patients without nephropathy.

**Methods:**

We prospectively enrolled 74 participants, including healthy volunteers (HVs), diabetic patients without nephropathy, and those with DN. The serum exosomal miRNA profiles of participants were examined using RNA sequencing.

**Results:**

The expression levels of 107 miRNAs differed between HVs and patients without DN, whereas the expression levels of 95 miRNAs differed between HVs and patients with DN. Among these miRNAs, we found 7 miRNAs (miR-1246, miR-642a-3p, let-7c-5p, miR-1255b-5p, let-7i-3p, miR-5010-5p, miR-150-3p) that were uniquely up-regulated in DN patients compared to HVs, and miR-4449 that was highly expressed in DN patients compared to patients without DN. A pathway analysis revealed that these eight miRNAs are likely involved in MAPK signaling, integrin function in angiogenesis, and regulation of the AP-1 transcription factor. Moreover, they were all significantly correlated with the degree of albuminuria.

**Conclusions:**

Patients with DN have a different serum exosomal miRNA profile compared to HVs. These miRNAs may be promising candidates for the diagnosis and treatment of DN and cardiovascular disease.

**Electronic supplementary material:**

The online version of this article (10.1186/s12967-019-1983-3) contains supplementary material, which is available to authorized users.

## Background

Diabetic nephropathy (DN) is one of the most important chronic complications found in patients with diabetes mellitus (DM) [1], and is a leading cause of end stage renal disease (ESRD) worldwide [2, 3]. Because of its increasing prevalence and the excessive risk of cardiovascular mortality, DN has become a burden to global public health [4]. Therefore, tremendous efforts have been made to understand the pathogenesis of DN and to identify therapeutic targets in order to develop new treatments for DN. Current treatments for DN include medications such as renin–angiotensin–aldosterone system inhibitors, sodium-glucose cotransporter 2 inhibitors, and glucagon-like peptide-1 receptor agonists that can reduce cardiovascular mortality and limit the progression of DN [5–7]. However, even the current best clinical practice for DN does not fully prevent DM patients suffering from complications and a progression to ESRD. Thus, further studies are warranted to unveil additional mechanisms to retard the progression of DN.

MicroRNAs (miRNAs) are endogenous, single-stranded, non-coding RNAs that regulate gene expression via post-transcriptional mechanisms. Currently, more than 2000 mature human miRNAs have been identified, and it is estimated that at least 60% of all human protein-coding genes are regulated by miRNAs [8]. Early studies examining the expression patterns of miRNAs in DN focused on miRNAs which were highly and specifically expressed in the kidney [9]. However, obtaining kidney tissues through a renal biopsy is highly invasive and hence these tissues sample are not readily available. Another important consideration is that DN is not only a kidney-specific disease, but also a consequence of the systemic complications of DM. Accordingly, some researchers have tried to investigate whether the levels of circulating miRNAs could be used for the early diagnosis and treatment of DN [10, 11]. However, the cell-free miRNAs present in circulation are highly heterogeneous. Cell-free miRNAs in the blood stream include the Ago2 protein bound form, the extracellular vesicle (EV)-enclosed form, and the vesicle-free form [12]. EV-incorporated miRNAs and whole cell-free miRNAs clearly differ from one another. miRNAs in EVs may have high chance to provide signatures reminiscent of their cell origin and are protected from the activity of extracellular RNases [13]. Furthermore, these exosome-enclosed miRNAs most likely function in intercellular communication and they could play a role in the pathology of various diseases [14–16]. To the best of our knowledge, there are few studies examining the circulating levels of exosomal miRNAs in patients with DN [17]. Moreover, all previous studies exploring the levels of miRNAs in DN have used real-time quantitative PCR (qPCR) [17]. However, because of the inherent limitations in this technique unidentified miRNAs are often not detected [18].

Here, we investigated circulating exosomal miRNA profiles in DM patients with DN, DM patients without nephropathy, and in heathy volunteers (HVs). The levels of miRNAs were compared by RNA sequencing in order to identify differentially expressed miRNAs that could highlight the potential pathways involved in the progression of DN in DM patients, as well as to possibly provide new targets for the treatment of DN.

## Methods

### Participants and data collection

We prospectively recruited participants who visited our center for a routine health check-up or for the treatment of DM at an outpatient clinic from January 2016 to August 2017. As a result, a total of 74 participants (18 healthy volunteers, 33 diabetic patients without nephropathy, and 23 patients with DN) who voluntarily provided informed consent were enrolled. This study was carried out in accordance with the Declaration of Helsinki, and the study protocol was approved by the institutional review board of Soonchunhyang University Seoul Hospital (2015-11-020).

All demographic data and anthropometric parameters were obtained at the time of enrollment. DM was defined according to World Health Organization criteria: fasting glucose ≥ 126 mg/dL, serum glucose ≥ 200 mg/dL after a 2 h oral glucose tolerance test, or a glycated hemoglobin (HbA1c) level ≥ 6.5%. Diabetic patients with an estimated glomerular filtration rate (eGFR) < 60 mL/min/1.73 m^2^ were not included in the study, because we focused on early DN. Therefore, patients with DN were defined as those who had history of DM and had showed micro- or macro-albuminuria (24-h urine albumin ≥ 30 mg/day or a spot urine albumin-to-creatinine ratio ≥ 30 mg/g) on at least three consecutive measurements. Blood samples were obtained at least 8 h after fasting, and an estimated glomerular filtration rate (eGFR) was calculated using the CKD Epidemiology Collaboration equation [19].

### Serum exosomal RNA isolation and assessment

The RNA sequencing process was conducted as described in previous study [20]. Briefly, circulating exosomes were isolated from serum using the ExoQuick isolation agent (System Bioscience, Palo Alto, CA, USA) according to the manufacturer’s guidelines. Supernatants, obtained after the centrifugation (3000×*g* for 15 min) of serum samples, were mixed with the ExoQuick reagent and incubated for 30 min at 4 °C. After another centrifugation at 1500×*g* for 30 min the supernatant was aspirated and the pellet retained. After resuspension of the pellet in sterile phosphate-buffered saline, the RNA was extracted using a miRNeasy Mini Kit (Qiagen, Hilden, Germany). All processes involving the suspension of exosomes were conducted according to the manufacturer’s guidelines. After RNA extraction, the purified RNA was eluted in RNase-free water (20 μL). The purified RNA was analyzed using an Agilent Bioanalyzer 2100 with an RNA Pico Chip and Small RNA Chip to examine the size distribution of the exosomal RNAs (Agilent Technologies, Santa Clara, CA, USA).

### cDNA library preparation and small RNA sequencing

The samples were processed to produce exosomal RNA (10 ng) as the input for each library. Small RNA libraries were constructed using a SMARTer smRNA-Seq Kit for Illumina^®^ (Takara Bio, Shiga, Japan) according to the manufacturer’s guidelines. Sequencing libraries were constructed by polyadenylation, cDNA synthesis, and PCR amplification.

The libraries were gel-purified and then validated by assessing their size, purity, and concentration using an Agilent Bioanalyzer. The libraries were quantified by qPCR according to the qPCR Quantification Protocol Guide (KAPA Library Quantification Kits for Illumina^®^ Sequencing Platforms). We assessed the quality of the libraries using a TapeStation D1000 ScreenTape (Agilent Technologies, Waldbronn, Germany). Equimolar amounts of the libraries were pooled and sequenced on an Illumina^®^ HiSeq 2500 instrument (Illumina, San Diego, CA, USA) to generate 101 base reads. Image decomposition and quality value calculations were performed using the modules in the Illumina^®^ pipeline. All procedures for next-generation sequencing (NGS) analysis were performed by Macrogen (Seoul, Korea).

### Analysis of RNA sequencing data and proportions of miRNAs

Following sequence alignment, known and novel microRNAs were identified using the miRDeep2 software algorithm. Prior to sequence alignment, we retrieved the *Homo sapiens* reference genome release hg19 from the UCSC Genome Browser, which we indexed using Bowtie (1.1.2), a program for aligning experimental and reference sequences. The reads were then aligned to the mature and precursor *H. sapiens* miRNAs obtained from miRBase 21. Uniquely clustered reads were sequentially aligned to the reference genome using miRBase 21 and the non-coding RNA database Rfam 9.1 to identify known miRNAs and other types of RNAs, respectively.

### Analysis of miRNA expression levels

The raw data (the reads for each miRNA) were normalized by relative log expression using DESeq 2. For pre-processing, miRNAs absent from more than 50% of all samples were excluded, leaving only mature miRNAs to be analyzed. We added 1 to the normalized read count of the filtered miRNAs to facilitate the log2 transformation to draw a correlation plot. For each miRNA, the baseMean and log fold change were calculated between groups. We conducted a statistical hypothesis test to compare two groups using the negative binomial Wald test in DESeq2. miRNAs differentially expressed between the two groups were defined as having a |fold change| ≥ 2 and a false discovery rate (FDR) adjusted *p* value of < 0.05. We also performed hierarchical clustering analysis using complete linkage and Euclidean distance as measures of similarity to display the expression patterns of the differentially expressed miRNAs that satisfied the criteria of a |fold change| ≥ 2 and an FDR adjusted p-value of < 0.05. All data analysis and visualization of the differentially expressed genes was conducted using R 3.3.1 (http://www.r-project.org).

### Identification of miRNA target genes and their molecular pathways

We uploaded the miRNAs that were differentially regulated in the normal healthy volunteers, the DM patients without nephropathy, and the DN groups, into commonly used analysis programs, such as DIANA-miRPath and miRSystem, for further analyses. The DIANA-miRPath v.3.0 database used DIANA-microT-CDS and TargetScan 6.2 to analyze the miRNA–gene interactions. The database schema incorporated the Kyoto Encyclopedia of Genes and Genomes (KEGG) pathways and the Gene Ontology (GO) and GO slim annotations. The gene and miRNA annotations were derived from Ensembl and miRBase, respectively. The miRSystem used seven algorithms for predicting miRNA targets (namely, DIANA-microT, miRanda, miRBridge, PicTar, PITA, RNA22, and TargetScan) and two experimentally validated databases of miRNA target genes (TarBase and miRecords). Five pathway databases, including GO, KEGG, BioCarta, Pathway Interaction Database, and Reactome, were used to annotate the biological functions and canonical pathways of the target genes.

### Statistical analyses

Continuous variables are expressed as the mean ± standard deviation, and categorical variables are expressed as a number and percentage. Non-normally distributed variables are expressed as a median and interquartile range. Comparisons between groups were conducted using a one-way analysis of variance, and a post hoc analysis between two groups was conducted using Bonferroni’s method. Comparisons between categorical and non-parametric variables were conducted using a Chi square test, and a Kruskal–Wallis test, as appropriate. Correlations between miRNAs levels and clinical parameters were assessed using a Spearman’s correlation analysis. Statistical analyses were conducted using SPSS version 23.0 (IBM Corporation, Armonk, NY, USA).

## Results

### Baseline clinical characteristics

The baseline clinical characteristics of the participants in this study are presented in Table 1. The mean age of the cohort was 45.9 years and 48.6% of participants were male. The HVs had no history of hypertension, cardiovascular disease, and smoking. Between the two groups of diabetic patients, only blood pressure was significantly higher in DM patients with nephropathy (systolic blood pressure, P = 0.038; diastolic blood pressure, P = 0.033) than in DM patients without nephropathy, whereas other characteristics were comparable between groups.

**Table 1:** Baseline characteristics of HVs and patients with DM

Variables	Group			Total (N = 74)	p-value
	HVs (N = 18)	DM without nephropathy (N = 33)	DM with nephropathy (N = 23)		
Age (years)	38.6 ± 8.0	49.2 ± 14.0	47.6 ± 14.7	45.9 ± 13.7	0.03
Sex (male, %)	5 (27.8)	18 (54.5)	13 (56.5)	36 (48.6)	0.124
HTN (n, %)	0 (0.0)	16 (48.5)	13 (56.5)	28 (37.8)	0.001
CVD (n, %)	0 (0.0)	2 (6.1)	4 (17.4)	6 (8.1)	0.135
Smoking (%)	0 (0.0)	14 (42.4)	12 (52.2)	27 (36.5)	0.007
BMI (kg/m^2^)	21.7 ± 1.4	29.8 ± 6.7	29.7 ± 7.6	27.7 ± 7.1	<0.001
SBP (mmHg)	106.1 ± 8.5	126.4 ± 11.5	134.4 ± 16.7	124.0 ± 16.5	<0.001
DBP (mmHg)	72.2 ± 8.1	77.4 ± 8.1	83.1 ± 11.5	78.1 ± 10.0	0.002
BUN (mg/dL)	11.6 ± 2.5	15.0 ± 4.0	17.5 ± 10.0	15.0 ± 6.6	0.016
Creatinine (mg/dL)	0.8 ± 0.1	0.8 ± 0.2	0.9 ± 0.4	0.8 ± 0.3	0.347
eGFR (mL/min/1.73 m^2^)	107.1 ± 10.9	100.7 ± 22.0	97.2 ± 29.0	101.5 ± 22.6	0.384
Hemoglobin (g/dL)	13.7 ± 1.0	13.8 ± 1.9	14.1 ± 2.2	13.9 ± 1.8	0.743
Fasting glucose (mg/dL)	97.0 ± 8.5	244.3 ± 219.5	219.5 ± 107.2	211.3 ± 133.1	< 0.001
Total protein (g/dL)	7.4 ± 0.3	7.1 ± 0.6	7.3 ± 0.6	7.3 ± 0.6	0.204
Serum albumin (g/dL)	4.7 ± 0.2	4.4 ± 0.3	4.5 ± 0.4	4.5 ± 0.3	0.003
Total cholesterol (mg/dL)	183.9 ± 24.2	177.2 ± 45.2	180.1 ± 49.2	179.9 ± 41.7	0.883
LDL cholesterol (mg/dL)	112.8 ± 23.0	112.9 ± 43.8	110.6 ± 44.1	112.4 ± 38.9	0.969
HDL cholesterol (mg/dL)	65.3 ± 14.6	44.9 ± 13.7	43.9 ± 13.4	49.4 ± 16.3	< 0.001
TG (mg/dL)	105.4 ± 82.9	203.0 ± 113.8	234.3 ± 215.7	188.5 ± 150.2	0.018
HbA1c (%)	–	9.9 ± 2.3	9.9 ± 2.3	10.0 ± 2.4	0.76
Albuminuria (mg/day)^a^	2.7 (1.7–4.2)	7.6 (4.4–10.1)	102.6 (36.3–408.1)	8.1 (3.5–28.9)	< 0.001

*HVs* healthy volunteers, *DM* diabetes mellitus, *HTN* hypertension, *CVD* cardiovascular disease, *SBP* systolic blood pressure, *DBP* diastolic blood pressure, *BUN* blood urea nitrogen, *eGFR* estimated glomerular filtration rate, *LDL* low density lipoprotein, *HDL* high density lipoprotein, *TG* triglyceride, *HbA1c* glycated hemoglobin

^a^Data are expressed as median and interquartile ranges

### Exosomal small RNA composition changes

To examine the composition of the exosomal small RNAs, we conducted NGS sequencing followed by mapping to each small RNA reference database. Using NGS, we identified exosomal small RNAs including miRNAs, small nuclear RNAs, small nucleolar RNAs, and transfer RNAs (tRNAs). More than half of the exosomal small RNAs were tRNAs and the proportion of small RNAs gradually increased when comparing HVs to DM patients with nephropathy. In diabetic patients, the proportion of miRNAs also tended to increase slightly (Additional file 1: Fig. S1).

### Profiles of exosomal miRNAs in DN

**Fig. 1:**
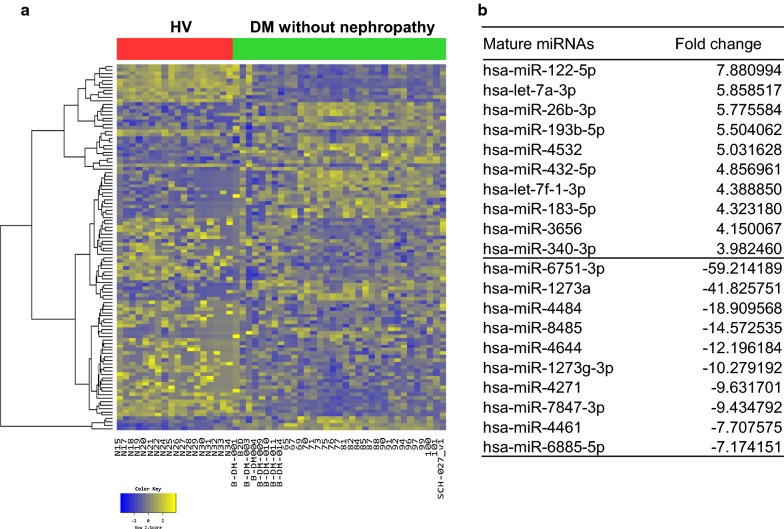
Exosomal miRNAs from patients with diabetes mellitus (DM) without nephropathy differ from those from healthy volunteers (HVs). **a** Heatmap showing Z-score of exosomal miRNAs from HVs (N = 18) and patients with DM without nephropathy (N = 33). 45 up-regulated (yellow) and 62 down-regulated (blue) miRNAs are presented. **b** Fold change of miRNAs expressions in patients with DM without nephropathy compared to HVs

**Fig. 2:**
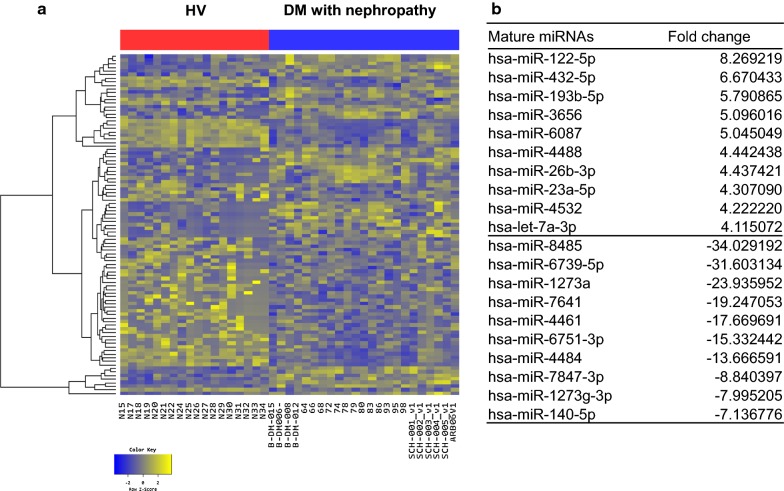
Exosomal miRNAs from patients with diabetes mellitus (DM) nephropathy differ from those from healthy volunteers (HVs). **a** Heatmap showing Z-score of exosomal miRNAs from HVs (N = 18) and patients with DM without nephropathy (N = 25). 40 up-regulated (yellow) and 55 down-regulated (blue) miRNAs are presented. **b** Fold change of miRNAs expressions in patients with DM nephropathy compared to HVs

**Fig. 3:**
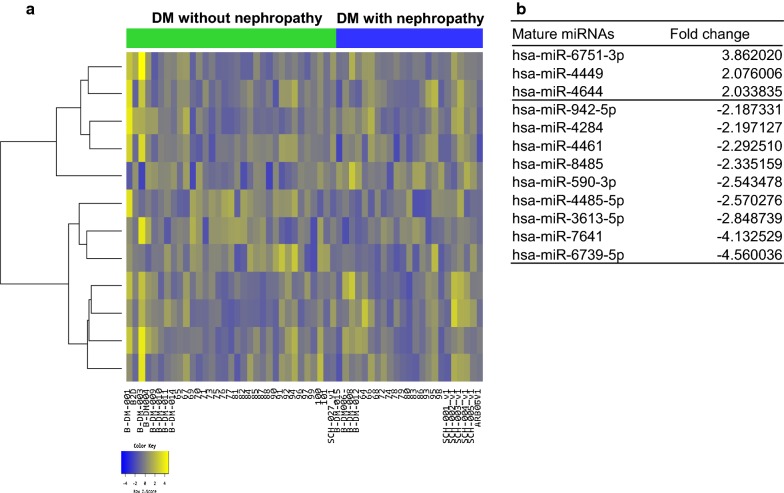
Exosomal miRNAs from patients with diabetes mellitus (DM) nephropathy differ from those from DM patients without nephropathy. **a** Heatmap showing Z-score of exosomal miRNAs from patients with DM nephropathy (N = 25) and those with DM without nephropathy (N = 33). 3 up-regulated (yellow) and 9 down-regulated (blue) miRNAs are presented. **b** Fold change of miRNAs expressions in patients with DM nephropathy compared to DM patients without nephropathy

After RNA sequencing, we identified 45 up-regulated and 62 down-regulated miRNAs in DM patients without nephropathy compared to HVs (Fig. 1). In addition, we also found 40 up-regulated and 55 down-regulated miRNAs in DN patients compared to HVs (Fig. 2). Between the two groups of diabetic patients, there were 3 up-regulated miRNAs and 9 down-regulated miRNAs in patients with DN compared to those without nephropathy (Fig. 3). To identify unique miRNAs in patients with DN, we selected eight miRNAs that were up-regulated only in patients with DN, and not in those without DN, compared to HVs (Additional file 1: Fig. S2). Among these eight miRNAs, miR-4449 was found to be up-regulated in common in patients with DN compared to both HVs and DM patients without nephropathy (Table 2). In addition, we evaluated the predicted biological pathways associated with these eight miRNAs using miRSystem. The possible pathways targeted by these miRNAs are presented in Table 3.

**Table 2:** Up-regulated circulating miRNAs only in patients with diabetic nephropathy

Mature miRNAs	Fold change	p-value
miR-4449	2.07	< 0.05
miR-1246	2.02	< 0.01
miR-642a-3p	2.51	< 0.05
let-7c-5p	2.49	< 0.001
miR-1255b-5p	2.08	< 0.01
let-7i-3p	2.36	< 0.01
miR-5010-5p	2.54	< 0.01
miR-150-3p	2.48	< 0.01

*miRNA* micro ribonucleic acid

**Table 3:** Top list of possible canonical pathways associated with 8 microRNAs which highly expressed in patients with diabetic nephropathy

Term	Total genes in the pathway	Union targets in the pathway	Score
MAPK signaling pathway	272	31	2.929
Intergrins in angiogenesis	74	13	2.134
AP-1 Transcription factor network	69	12	1.971
Olfactory transduction	388	1	1.926
Olfactory signaling pathway	377	1	1.863
NCAM1 interactions	44	9	1.752
Signaling by PDGF	122	15	1.702
Axon guidance	266	24	1.701
TNF receptor signaling pathway	46	9	1.692
NCAM signaling for neurite out-growth	70	11	1.671
IL-4 mediated signaling events	64	10	1.533
Developmental biology	494	35	1.533
SHP2 signaling	54	9	1.484
Beta3 integrin cell surface interactions	43	8	1.47
Cell cycle	124	14	1.461
C-myb transcription factor network	81	11	1.456
P53 signaling pathway	68	10	1.451
TGF-β signaling pathway	84	11	1.405
Syndecan-1 mediated signaling events	46	8	1.392
Ion transport by p-type ATPases	36	7	1.358

*miRNA* micro ribonucleic acid

### Relationship between exosomal miRNAs and clinical parameters

A Spearman’s correlation coefficient was used to examine the correlation between miRNAs and clinical parameters (Table 4). As a result, a significant association with HbA1c was only found for miR1255b-5p, and no correlation with eGFR was seen for any of the miRNAs. However, we did identify a significant correlation with the degree of albuminuria for all eight miRNAs.

**Table 4:** Correlation between miRNAs and clinical parameters

Mature miRNAs	Age	eGFR	BMI	MAP	Glucose	HbA1c	HDL	TG	Albuminuria
miR-1246									
γ	0.019	− 0.066	0.416	0.135	0.120	− 0.143	− 0.195	0.387	0.373
p-value	0.876	0.576	< 0.001	0.250	0.309	0.299	0.109	0.001	0.001
miR-642a-3p									
γ	0.039	− 0.099	0.106	0.100	0.209	0.014	− 0.121	0.194	0.362
p-value	0.740	0.404	0.367	0.398	0.074	0.918	0.314	0.105	0.002
let-7c-5p									
γ	0.420	− 0.091	0.184	0.331	0.374	0.016	− 0.223	0.281	0.428
p-value	< 0.001	0.443	0.117	0.004	0.001	0.907	0.062	0.018	<0.001
miR-1255b-5p									
γ	0.166	0.219	0.176	0.133	0.478	0.318	− 0.421	0.346	0.240
p-value	0.157	0.061	0.135	0.258	< 0.001	0.018	< 0.001	0.003	0.039
let-7i-3p									
γ	0.129	− 0.125	0.086	0.101	0.078	− 0.265	− 0.125	0.022	0.300
p-value	0.273	0.287	0.467	0.390	0.510	0.050	0.299	0.859	0.009
miR-5010-5p									
γ	0.211	− 0.023	0.232	0.313	0.337	0.011	− 0.172	0.306	0.254
p-value	0.070	0.845	0.047	0.007	0.003	0.936	0.150	0.009	0.029
miR-150-3p									
γ	0.154	− 0.078	0.194	0.041	0.213	− 0.039	− 0.131	0.062	0.347
p-value	0.189	0.507	0.098	0.729	0.068	0.777	0.277	0.608	0.002
miR-4449									
γ	− 0.015	− 0.007	0.385	0.161	0.191	− 0.237	− 0.335	0.268	0.497
p-value	0.899	0.953	0.001	0.169	0.103	0.081	0.004	0.024	< 0.001

*miRNA* micro ribonucleic acid, *eGFR* estimated glomerular filtration rate, *BMI* body mass index, *MAP* mean arterial pressure, *HbA1c* glycated hemoglobin, *HDL* high density lipoprotein, *TG* triglyceride

## Discussion

In this study, we conducted RNA sequencing to examine the profile of circulating exosomal miRNAs in patients with DN. As a result, we identified eight DN-specific miRNAs whose levels were up-regulated in circulating exosomes. In addition, the levels of these miRNAs were found to be significantly correlated with the degree of albuminuria. To our knowledge, this is the first study to examine miRNA profiles specifically in circulating exosomes in patients with DN.

Several previous studies have tried to characterize the circulating miRNAs in patients with DN [21–27]. However, the miRNAs found in this study did not overlap with the miRNAs found in these previous studies. There are several possible explanations for this. First, all of the previous studies conducted used RNA isolation and quantification from whole serum or plasma, but we specifically measured RNA expression levels in circulating exosomes. The progression of diabetes is a consequence of pathological alterations in several tissues such as the liver, skeletal muscle, and adipose tissue, which can cause glucose intolerance, β-cell dysfunction, and the development of complications [28]. Therefore, inter-organ communications are important for the development of diabetes. Many previous studies have reported diverse circulating factors including hormones, cytokines, and growth factors that can modulate inter-organ communications in diabetes [29]. Moreover, recent studies have also revealed that circulating exosomal miRNAs can regulate gene expression in distant organs [30, 31]. Therefore, circulating exosomal miRNAs, rather than non-specific circulating miRNAs, may be more reliable candidates to explore the underlying disease mechanisms in patients with DN. Second, in all the previous studies RNA quantification was carried out using qPCR and microarrays, whereas we used RNA sequencing in this study. While RNA sequencing is not a replacement for other RNA quantification methods it is complementary to them. However, RNA sequencing has the advantages of highly reproducibility, low background, large dynamic range, and the ability to perform untargeted analysis [18, 32]. Therefore, for this study we believed that it was better to use RNA sequencing to discover unknown circulating exosomal miRNAs associated with DN [18]. Third, the different characteristics of patients may affect the different miRNA profiles. Some of the previous studies conducted their analysis using type 1 diabetic patients [23, 26], whereas other studies enrolled only diabetic patients, but not HVs [21, 27, 33]. Because of the excessive cardiovascular risk, the survival of DN patients is dramatically reduced as renal function declines [34]. However, the renal damage in diabetes forms a part of the systemic vascular complications that arise in this disease. A previous study has reported that the development of microalbuminuria, an early marker of DN, is a valid prognostic factor that can be used to predict future cardiovascular events [35]. Therefore, it is important to evaluate the unique characteristics of diabetic patients with vascular complications compared to those patients without vascular complications and healthy population. Accordingly, we selected seven miRNAs that were significantly up-regulated in DM patients with DN when compared to either HVs or to DM patients without nephropathy. In addition, one miRNA (miR-4449) was found to be commonly up-regulated in DN patients when compared to both HVs and DM patients without nephropathy. Moreover, the expression levels all of these miRNAs were found to be significantly correlated with the degree of albuminuria.

We found that the miRNA let-7c-5p was significantly up-regulated in patients with DN when compared to HVs. Previous studies have reported that the miRNA let-7 family members (let-7b and let-7c) play functional roles in renal fibrosis in DM through the regulation of transforming growth factor-β (TGF-β) signaling [36, 37]. In addition, one study also reported that mesenchymal stem cells, engineered to overexpress and deliver the miRNA let-7c via exosomes, attenuated renal fibrosis via the TGF-β1 pathway [38]. Therefore, it is likely that the up-regulated levels of let-7c-5p in our study are a physiological response to counteract the renal fibrosis seen in DN, and so let-7c may be a potential therapeutic target for the diagnosis and treatment of DN. Meanwhile, Pezzolesi et al. reported contradictory results that decreased circulating let-7c-5p was associated with increased risk of ESRD in type 1 diabetes patients that had been treated for more than 20 years [23]. Considering that TGF-β1 signaling also suppressed let-7c expression [37], subjects in the study of Pezzolesi et al. might already have prevalent renal fibrosis with highly activated TGF-β signaling, which can lead to down-regulated let-7c-5p. Further research is needed to describe the interaction between let-7c and TGF-β1 in progression of DN. The miRNA miR-150 was also evaluated in a previous study by Ranganathan et al. who reported that the deletion of miR-150 is associated with the suppression of inflammation and apoptosis in endothelial cells in the kidney seen as a result of myocardial infarction [39]. Interestingly, the downregulation of platelet miR-150 has also been reported in patients with atrial fibrillation (AF) and chronic heart failure [40]. AF increases the risk of stroke or mortality. Thus, the dysregulation of miR-150 may be one of the regulators that mediates cardiovascular damage. miR-642a-3p has also previously been reported to be a key regulator of adipogenesis [41]. Using deep sequencing, miR-642a-3p was shown to be significantly up-regulated during the adipogenic differentiation of human adipose tissue-derived stem cells. Adipocytes are not simply spaces for storing fat, but they also exert systemic metabolic effects by secreting adipokines via exosomes and extracellular vesicles [42], and are a main source of exosomal miRNAs [31]. Therefore, miR-642a-3p, which was up-regulated in patients with DN in our study, may a candidate for the inter-organ communication between adipose tissue and the kidney in diabetic patients. Another recent study reported that the levels of let-7i-3p in urinary extracellular vesicles was significantly increased in patients with DN, and is associated with degree of albuminuria [43]. Considering that let-7i-3p was also up-regulated in circulating exosomes and correlated with albuminuria in our study, increased let-7i-3p levels in serum and urine may indicate early progression in DN. The other miRNAs we found have not previously been reported to be associated with metabolic disease, and therefore further studies are warranted in order to understand the relationship between these miRNAs and DN.

We also analyzed the canonical biological pathways associated with the eight miRNAs we identified using miRSystem and DIANA-miRPath. These pathways have previously been reported to be involved in the pathogenesis of diabetic complications. The mRNA levels of mitogen-activated protein kinase (MAPK) signaling pathway components have been shown to be significantly increased in the kidney of diabetic patients compared to healthy population [44], and reduced levels of p38 MAPK signaling have been found to be associated with protection against renal injury and this signaling pathway plays a distinct pathogenic role in the progression of DN [45]. In addition, the integrin-vascular endothelial growth factor axis is a key factor in pathological angiogenesis including tumor metastasis, tissue remodeling, and diabetic complications [46]. Activator protein-1, along with nuclear factor-κB, has been reported to be associated with the increased release of inflammatory cytokines and the activation of monocytic cells in DN [47, 48]. Moreover, the olfactory receptor is expressed in pancreatic β-cells and renal tubules, and modulates systemic glucose metabolism, renin secretion, and GFR [49, 50]. Further studies are needed to evaluate the association between these miRNAs and the biological pathways we have identified, and it is hoped that this may reveal the hidden mechanisms underlying the progression of DN.

There are several limitations in our study that should be discussed. First, a relatively small number of participants were enrolled in this study; further studies with a larger cohort are needed to confirm our findings. Second, some demographic factors and clinical parameters were significantly different between groups, and these factors might affect circulating exosomal miRNA profiles. Third, although we conducted exosomal RNA sequencing using validated commercial kits, the procedures used to isolate exosomes are not standardized and a systematic bias in library preparation may lead to the false-positive identification of miRNAs [18, 51]. Further research with complementary methods such as qPCR is therefore needed to validate the novel miRNAs we identified. Fourth, our raw RNA sequencing data were normalized using DESeq 2, but another statistical approach may more accurately assess RNA expression level. Fifth, although we showed correlation between clinical parameters and the novel miRNAs we identified in DN, we only could suggest potential molecular pathways via bio-informatic analysis. Future experimental studies directly testing the relationships between miRNAs and clinical parameters are warranted. Finally, the circulating exosomes we isolated are likely to be derived from a various cell populations and we are therefore unable to investigate the true origin of the exosomes we isolated. It is likely that the exosomes shed from different cells have different miRNA profiles.

## Conclusions

We identified a unique profile of circulating exosomal miRNAs in patients with DN. Our findings might form the basis for uncovering the inter-organ communication and pathogenesis in the progression of various diabetic complications including DN. Therefore, further studies examining the function of these miRNAs are warranted to understand the physiological mechanisms occurring in DN and to improve the outcomes in these patients.

## Additional file

**Additional file 1: Figure S1.** Small RNA composition changes in circulating exosomes by RNA sequencing. *P < 0.05 vs. healthy volunteers, ^†^P < 0.05 vs. DM without nephropathy.

## Data Availability

The data that support the findings of this study are available from the corresponding author upon reasonable request.

## Abbreviations

DN: diabetic nephropathy
ESRD: end stage renal disease
miRNA: microRNA
EV: extracellular vesicle
qPCR: quantitative polymerase chain reaction
HV: healthy volunteer
HbA1c: glycated hemoglobin
eGFR: estimated glomerular filtration rate
NGS: next-generation sequencing
FDR: false discovery rate
KEGG: Kyoto Encyclopedia of Genes and Genomes
GO: Gene Ontology
tRNA: transfer RNA
TGF-β: transforming growth factor-β
AF: atrial fibrillation
MAPK: mitogen-activated protein kinase
