# Supplementary material for: The circulating exosomal microRNAs related to albuminuria in patients with diabetic nephropathy
Source: J Transl Med. 2019 Jul 22;17:236. doi: 10.1186/s12967-019-1983-3 (PMC6647278; doi:10.1186/s12967-019-1983-3)
Supplement: Supplementary file 1 — Additional file 1: Figure S1. Small RNA composition changes in circulating exosomes by RNA sequencing. *P < 0.05 vs. healthy volunteers, †P < 0.05 vs. DM without nephropathy. [file 12967_2019_1983_MOESM1_ESM.pdf]

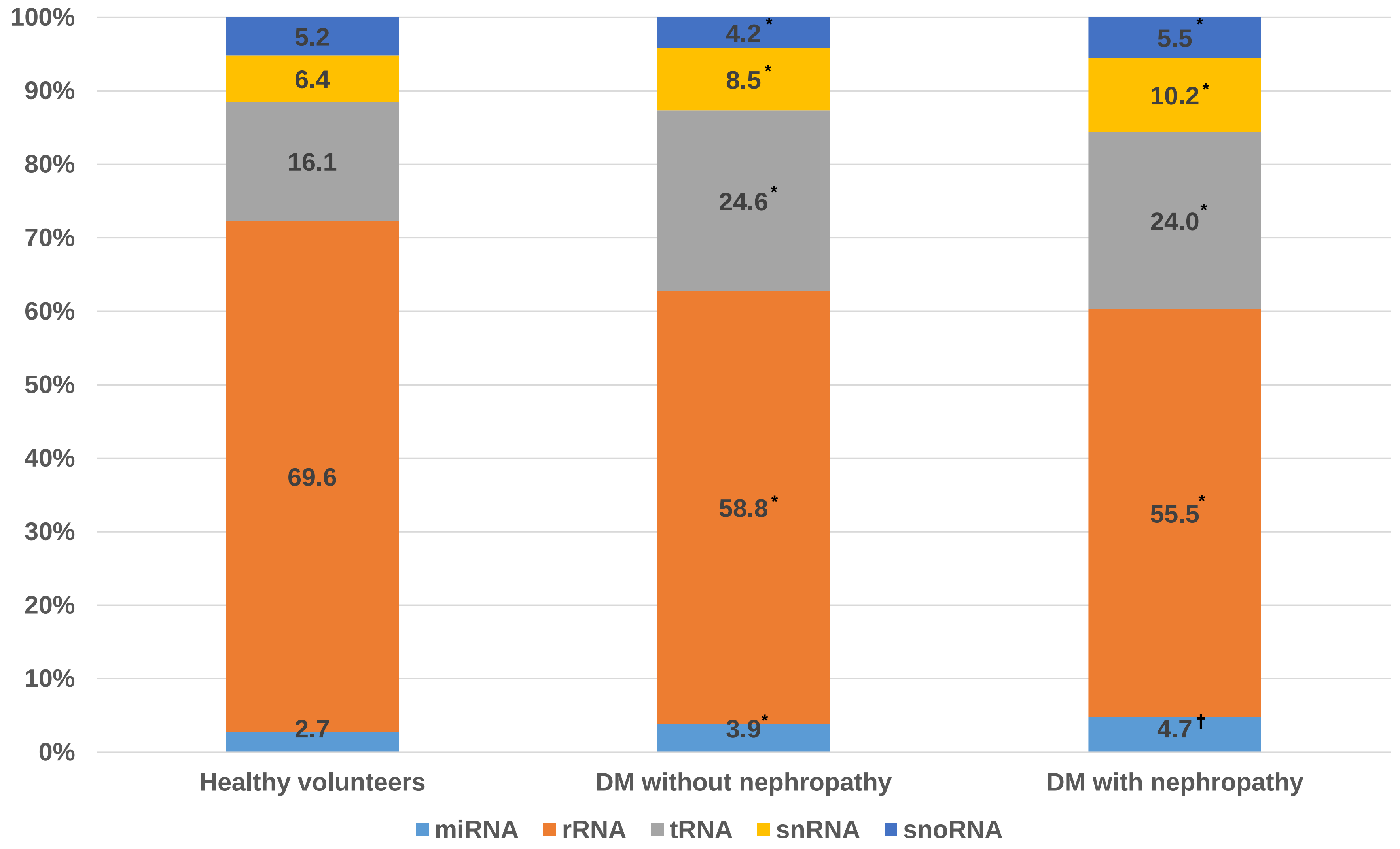

Figure S1. Small RNA composition changes in circulating exosomes by RNA sequencing.
***P<0.05 vs. healthy volunteers, †P<0.05 vs. DM without nephropathy**

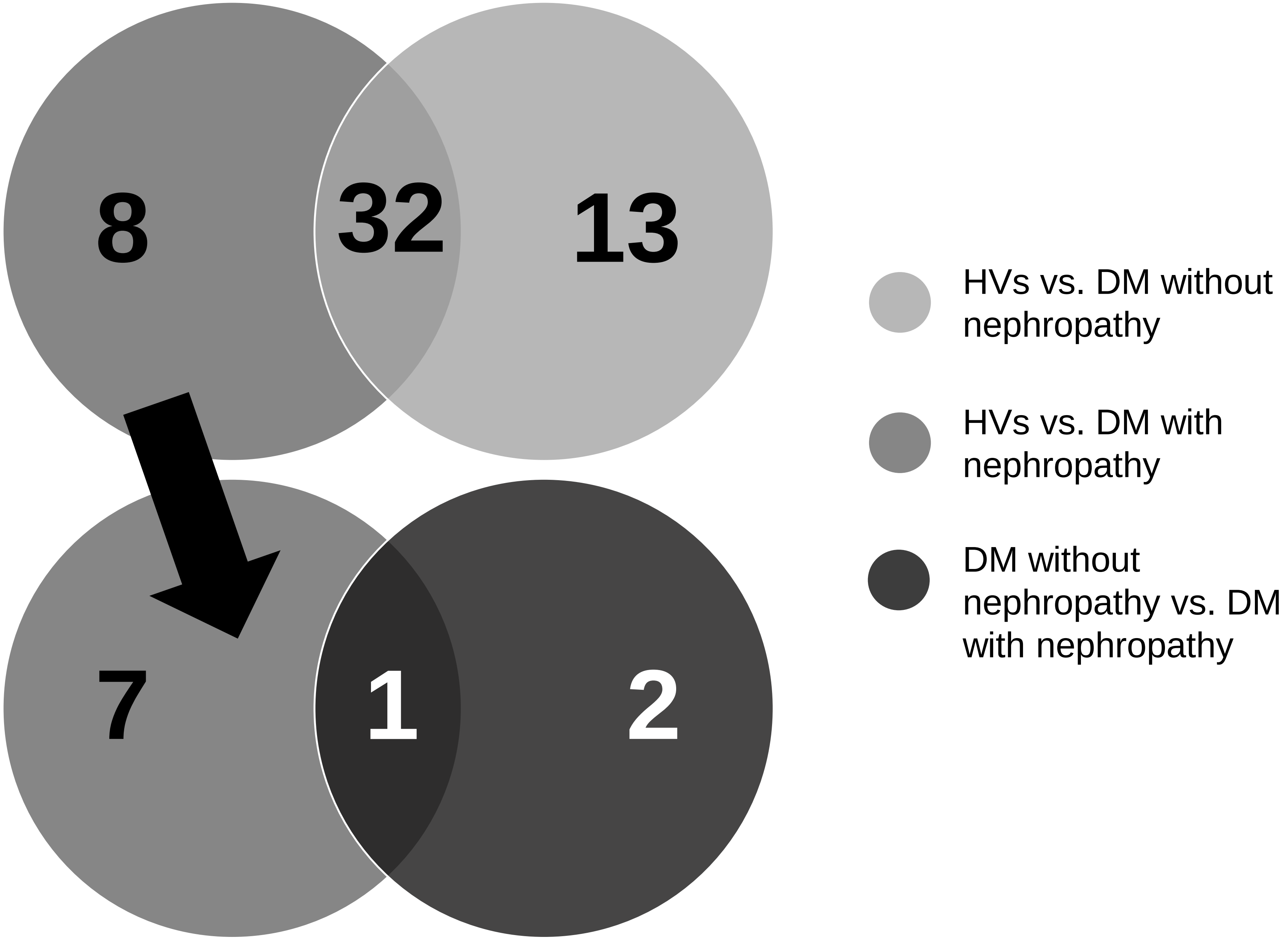

Figure S2. Selection process of miRNAs uniquely up-regulated in patients with diabetes mellitus (DM) nephropathy
